# Effect assessment of methotrexate in combination with other chemotherapeutic agents for osteosarcoma in children: A protocol for systematic review and meta-analysis

**DOI:** 10.1097/MD.0000000000025534

**Published:** 2021-05-21

**Authors:** Jun-Ping Yan, Rong-Mei Xiang

**Affiliations:** Department of Pediatrics, Hanchuan People's Hospital, Hanchuan, Hubei, China.

**Keywords:** chemotherapy, meta-analysis, methotrexate, osteosarcoma, protocol

## Abstract

**Background:**

Osteosarcoma is a primary form of malignant bone tumor. It is commonly prevalent among children. Treating osteosarcoma with chemotherapy has had limited clinical outcomes due to side effects and the formation of drug resistance. Presently, a mixture of doxorubicin, cisplatin, ifosfamide, epirubicin methrotrexate, and other supplementary medications are used in osteosarcoma chemotherapy. Therefore, this study aims to investigate the clinical therapeutic effects of combining methotrexate with other chemotherapeutic agents to treat osteosarcoma in children.

**Methods:**

The search of several electronic databases will lead to source related published studies. The electronic databases include both English (PubMed, EMBASE, Web of Science, and the Cochrane Library) and Chinese (China National Knowledge Infrastructure, WanFang, and China Biomedical Database) databases. All studies published from inception to November 19, 2020 are searched. Study selection, extraction of data, and evaluation of the bias risk in included studies are carried out by two authors independently. The software, RevMan 5.3, is used to analyze the data.

**Results:**

This study provides evidence of substantial quality for the clinical therapeutic effects of methotrexate combined with other chemotherapeutic agents for treating osteosarcoma in children.

**Conclusion:**

The results of this study provide conclusive evidence with regards to the clinical application of methotrexate combined with other chemotherapeutic agents for treating osteosarcoma in children.

**Ethics and dissemination:**

Since this study will use published data, ethical approval is not required.

**Systematic review registration number:**

This protocol has been registered on INPLASY202110024.

## 1 Introduction

Osteosarcoma is a primary form of malignant bone tumor. It is commonly prevalent among children. Annually, an estimated 4.7 individuals per million individuals aged <20 years are diagnosed with osteosarcoma.^[1,2]^ Overall, the 5-year survival rate of osteosarcoma patients is roughly 68% percent. However, depending on the stage of diagnosis, the actual number ranges from 40% to 80%.^[3]^ Technological advancements have made significant breakthroughs in the field of neoadjuvant chemotherapy, resulting in significantly improving the positive clinical outcomes and potentiated local treatment with the salvation and reconstruction of limbs.^[4]^ Admittedly, the survival rate of osteosarcoma patients has improved over the years. However, its etiology is yet to be defined.^[5,6]^ Past studies have attempted to elucidate the etiology of osteosarcoma. These studies involve epidemiologic and environmental factors, as well as genetic impairments.^[5,6]^

Chemotherapy is a crucial therapeutic method for treating cancer. A sufficient dose of intracellular chemodrugs should be delivered to the cancer site to kill cancer cells and treat cancers effectively.^[7]^ There are 4 main chemodrugs, namely, high-dose methotrexate, cisplatin, doxorubicin, and ifosfamide.^[8,9]^ Existing treatment protocols primarily combine ≥2 of these 4.^[8,9]^ In recent years, methotrexate has indicated to be the most active drug. However, at the moment, it is unknown whether it is essential to improve the survival rate of children diagnosed with osteosarcoma. The present systematic review and meta-analysis are conducted to explore the clinical effects of combining methotrexate with other chemotherapeutic agents to treat children diagnosed with osteosarcoma.

## 2 Objectives

The aim of this study was to systematically evaluate the clinical therapeutic effects of methotrexate combined with other chemotherapeutic agents to treat osteosarcoma in children. It is also expected to provide reference for chemotherapy in children diagnosed with osteosarcoma.

## 3 Methods

The present systematic review protocol is registered on the International Platform of Registered Systematic Review and Meta-analysis Protocols (INPLASY) platform with the identification number INPLASY202110024. This protocol will be performed and reported strictly based on the guidelines of the Preferred Reporting Items for Systematic Review and Meta-Analyses Protocols (PRISMA-P) statement.

## 4 Eligibility criteria

### 4.1 Types of studies

It is intended to collect randomized controlled trials (RCTs) on methotrexate combined with other chemotherapeutic agents in children diagnosed with osteosarcoma.

### 4.2 Types of participants

Children (aged <15 years) diagnosed with osteosarcoma who received treatment with methotrexate-based combinational chemotherapy will be enrolled.

### 4.3 Types of interventions and comparisons

The treatment of methotrexate-based combinational chemotherapy was administered to the experimental group. Meanwhile, the control group was treated without methotrexate-based chemotherapy

### 4.4 Types of outcome measures

#### 4.4.1 Major outcomes

The major outcomes are overall survival rate (the time taken to die from any cause); relapse-free survival rate (the time taken for the recurrence of osteosarcoma following surgery); response rate (denotes the classical response rates).

#### 4.4.2 Minor outcomes

The minor outcomes are toxicities; quality of life; adverse events, including gastrointestinal manifestations and hepatotoxicity.

## 5 Data sources and search

A systematic search of the electronic databases given below from their beginning to November 19, 2020 will be conducted: English databases include PubMed, EMBASE, Web of Science, and the Cochrane Library. The Chinese databases include China National Knowledge Infrastructure, WanFang, and China Biomedical Database. The language restrictions were English and Chinese. The following MESH terms and related synonyms, including osteosarcoma∗, chemotherapy∗, methotrexate∗, “randomized controlled trial”, “randomised controlled trial”, randomly∗, and RCT∗ were combined in the search strategy to search the databases mentioned above.

## 6 Data collection and analysis

### 6.1 Study selection

**Figure 1:**
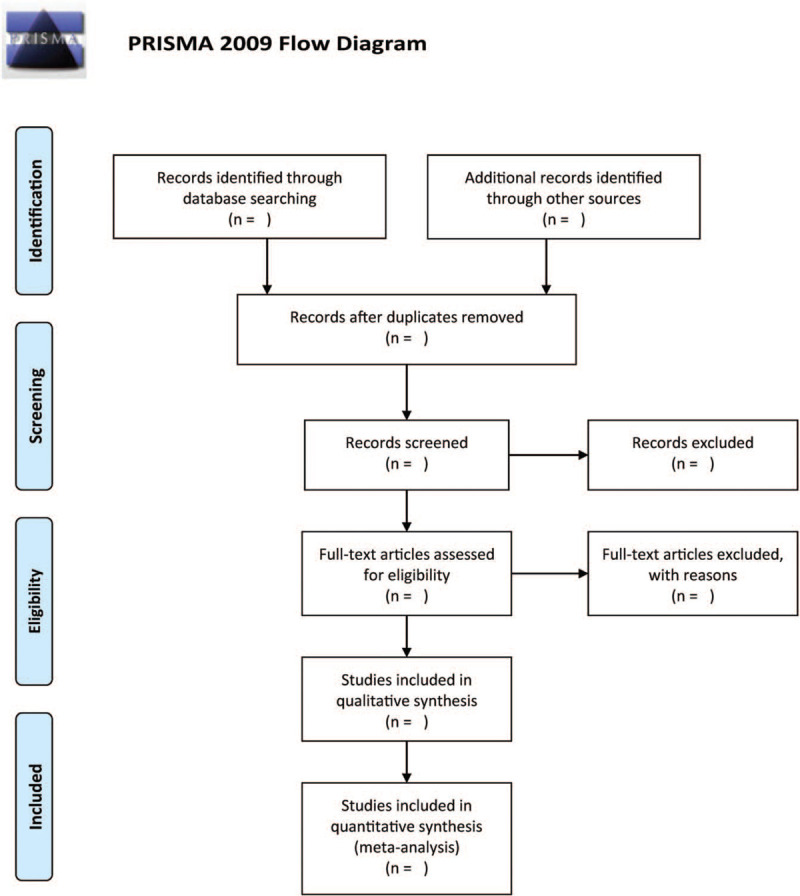
The flow diagram of the study selection process.

The screening of the titles/abstracts in each potential study is done independently by two authors. Following this, unrelated studies will be removed. The full text of the relevant studies will be read to assess the literature based on the criterion for including and excluding studies. All disagreements will be resolved through discussion. The process of searching and screening literature will be presented in Figure 1.

### 6.2 Data extraction and management

Two authors will use EndNote X9.0 software to manage the literature. They will plan to independently extract the following information into the pre-designed Excel 2019 table: first author's name, publication date, country, study method, sample size, sex ratio, mean age, intervention and comparison measures, dosage, number of response and nonresponse events, duration of therapy, follow-up time, and adverse events. All disagreements will be resolved through discussion.

### 6.3 Assessment of risk of bias in included studies

Two authors will evaluate the assessment of risk of bias based on the Cochrane Risk of Bias Tool. According to these criteria (generation of random sequence, allocation concealment, blind method, incomplete data, selective reporting outcomes, and additional biases), risk of bias will be classified into three levels: “low risk,” “high risk,” or “unclear.” All disagreements will be resolved through discussion.

### 6.4 Measures of treatment effect

Relative risk with 95% confidence interval (CI) will be used to calculate the summary statistic for dichotomous variables. Meanwhile, the mean difference or standardized mean difference with 95% CI will be utilized to calculate as the summary statistic for continuous variables.

### 6.5 Dealing with missing data

In the event where data are missing or insufficient, the corresponding author will be contacted to obtain the data. If not, the incomplete study will be removed.

### 6.6 Assessment of heterogeneity

The heterogeneity will be assessed by *χ*^2^ and *I*^2^ values. Obvious heterogeneity is not present when *I*^2^ > 50% or *P* < .1, in which case the random-effects model will be used^[10]^; otherwise, the fixed-effects model will be used.^[11]^

### 6.7 Assessment of reporting biases

Funnel plots and Egger test will be used to evaluate potential reporting biases if the numbers of available studies are sufficient.

### 6.8 Sensitivity analysis

Sensitivity analysis will be used by sequentially eliminating one study at a time to determine the stability of our findings.

### 6.9 Subgroup analysis

If possible, subgroup analysis will be performed based on the different study characteristics, age, sex, ethnicity, and dosage.

## 7 Discussion

Osteosarcoma, a rare type of sarcoma, is the most prevalent form of malignant bone tumor in children.^[12]^ Admittedly, published studies have reported that methotrexate has been used for the treatment of osteosarcoma. However, it has not been reported whether it is essential for improving the survival for osteosarcoma in children. Therefore, the present systematic review and meta-analysis will help identify the potential clinical therapeutic effects of methotrexate in treating children diagnosed with osteosarcoma. The present study will provide reference for chemotherapy in children diagnosed with osteosarcoma.

## Author contributions

**Conceptualization:** Rongmei Xiang.

**Data curation:** Jun-Ping Yan, Rongmei Xiang.

**Formal analysis:** Jun-Ping Yan, Rongmei Xiang.

**Investigation:** Jun-Ping Yan.

**Methodology:** Jun-Ping Yan, Rongmei Xiang.

**Project administration:** Jun-Ping Yan.

**Resources:** Rongmei Xiang.

**Software:** Jun-Ping Yan.

**Supervision:** Rongmei Xiang.

**Validation:** Jun-Ping Yan.

**Visualization:** Jun-Ping Yan.

**Writing – original draft:** Jun-Ping Yan, Rongmei Xiang.

**Writing – review & editing:** Rongmei Xiang.
